# Ecosystem Services Modeling as a Tool for Defining Priority Areas for Conservation

**DOI:** 10.1371/journal.pone.0154573

**Published:** 2016-05-04

**Authors:** Gabriela Teixeira Duarte, Milton Cezar Ribeiro, Adriano Pereira Paglia

**Affiliations:** 1 Laboratório de Ecologia e Conservação, Departamento de Biologia Geral, Instituto de Ciências Biológicas, Universidade Federal de Minas Gerais, Belo Horizonte, Minas Gerais, Brazil; 2 Laboratório de Ecologia Espacial e Conservação (LEEC), Departamento de Ecologia, Universidade Estadual Paulista Julio de Mesquita (UNESP), Rio Claro, São Paulo, Brazil; Universidade Federal de Goiás, BRAZIL

## Abstract

Conservationists often have difficulty obtaining financial and social support for protected areas that do not demonstrate their benefits for society. Therefore, ecosystem services have gained importance in conservation science in the last decade, as these services provide further justification for appropriate management and conservation of natural systems. We used InVEST software and a set of GIS procedures to quantify, spatialize and evaluated the overlap between ecosystem services—carbon stock and sediment retention—and a biodiversity proxy–habitat quality. In addition, we proposed a method that serves as an initial approach of a priority areas selection process. The method considers the synergism between ecosystem services and biodiversity conservation. Our study region is the Iron Quadrangle, an important Brazilian mining province and a conservation priority area located in the interface of two biodiversity hotspots, the Cerrado and Atlantic Forest biomes. The resultant priority area for the maintenance of the highest values of ecosystem services and habitat quality was about 13% of the study area. Among those priority areas, 30% are already within established strictly protected areas, and 12% are in sustainable use protected areas. Following the transparent and highly replicable method we proposed in this study, conservation planners can better determine which areas fulfill multiple goals and can locate the trade-offs in the landscape. We also gave a step towards the improvement of the habitat quality model with a topography parameter. In areas of very rugged topography, we have to consider geomorfometric barriers for anthropogenic impacts and for species movement and we must think beyond the linear distances. Moreover, we used a model that considers the tree mortality caused by edge effects in the estimation of carbon stock. We found low spatial congruence among the modeled services, mostly because of the pattern of sediment retention distribution.

## Introduction

Ecosystem services are the benefits that natural ecosystems provide for humans [1–3]. Among them are the provision of food, wood, water quality, climate regulation, wildlife-based tourism and pollination of crops. This concept has garnered great importance in conservation science in the last decade [4]. The Millennium Ecosystem Assessment has reported a widespread decline in ecosystem services across the world [3]. This research emphasized the urgent need to incorporate services into the decision-making process in order to ensure human well-being, presently and in the future. In this context, the Conference of Parties (COP 10) to the Convention on Biological Diversity (CBD) established a global strategic plan for biodiversity in which, among others, the protection and the restoration of ecosystem services are targets to be accomplished by 2020 [5].

Conservationists often have difficulty obtaining financial and social support for protected areas that do not demonstrate their benefits for society [6,7]. Normally, they define as priority areas those that are rich in species, concentrate high levels of endemism [8–10], and exist within a well-connected and highly conserved context [11]. In most cases, the development and implementation of this prioritization strategy is unrelated to the economic and social debate. However, the integration of these strategies and debates could reduce the conflicts and trade-offs among them [12]. The ecosystem services approach supports biodiversity conservation, because services provide further justification for appropriate management and conservation of natural systems as well as for more financial support for these two activities [6,13]. In this sense, the ecosystem services approach has the potential to preserve areas outside legally protected reserves, which is an important feature amid the global proliferation of disturbed landscapes [3,6,14,15]. These areas are usually maintained through payments for environmental services (PES), in which beneficiaries pay landowners for the conservation and maintenance of ecosystems and their services [16–18]. This is a promising way to align social and economic development with protection of natural environments and their ecological processes.

The modeling and mapping of ecosystem services are important elements in a decision-making process that aims to improve recognition and application of services [19]. Spatial prioritization is also considered an important step in conservation planning [20]. With spatial and quantitative information, land use decisions could incorporate areas with the best trade-offs and win-wins between services, biodiversity conservation and economic activities [21]. Those are very important tools for decision-making, especially in conflict regions, where the economic activities affect the natural surroundings. According to Seppelt *et al*. [22], the recent studies on mapping and quantifying ecosystem services are concentrated in a few countries (50% of 153 reviewed works are located in only six countries) and are lacking in research on tropical areas. However, habitat loss and the consequent biodiversity loss is a global problem, and it is more prominent in the tropics [3,23]. Moreover, the works that sought to analyze the overlap between biodiversity-rich areas and areas providing services are still incipient and have conflicting results [15,21,24–26]. This suggests a need to extend this kind of research, mostly in places where current human activity can harm the conservation of the natural capital.

Here, we take as a region of study the Iron Quadrangle, located in southeastern Brazil. Besides being an important mineral reserve for the country [27], the Iron Quadrangle is also a conservation priority area [28]. Located in the interface of two Brazilian biodiversity hotspots, Atlantic Forest and Cerrado [9], the Iron Quadrangle has a high endemism level of amphibians and plants, high vertebrates richness, a large extension of ironstone outcrops—one of the country’s most threatened geologic formations [28,29]—and important groundwater and watersheds for human population.

Therefore, the region’s biodiversity, endemism, human demand for services and economic pressures on the environment illustrate the need to incorporate human well-being and economic externalities into conservation science. Focusing on habitat quality (a biodiversity indicator) and the ecosystem services carbon stock and sediment retention, the aims of this work were to: 1) quantify and spatialize these ecosystem services and indicator; 2) identify which parameters influence the ecosystem services models; 3) evaluate the overlap and the synergism between ecosystem services and biodiversity indicator; 4) indicate priority areas for ecosystem services and biodiversity conservation.

## Methodology

### Study area

**Fig 1:**
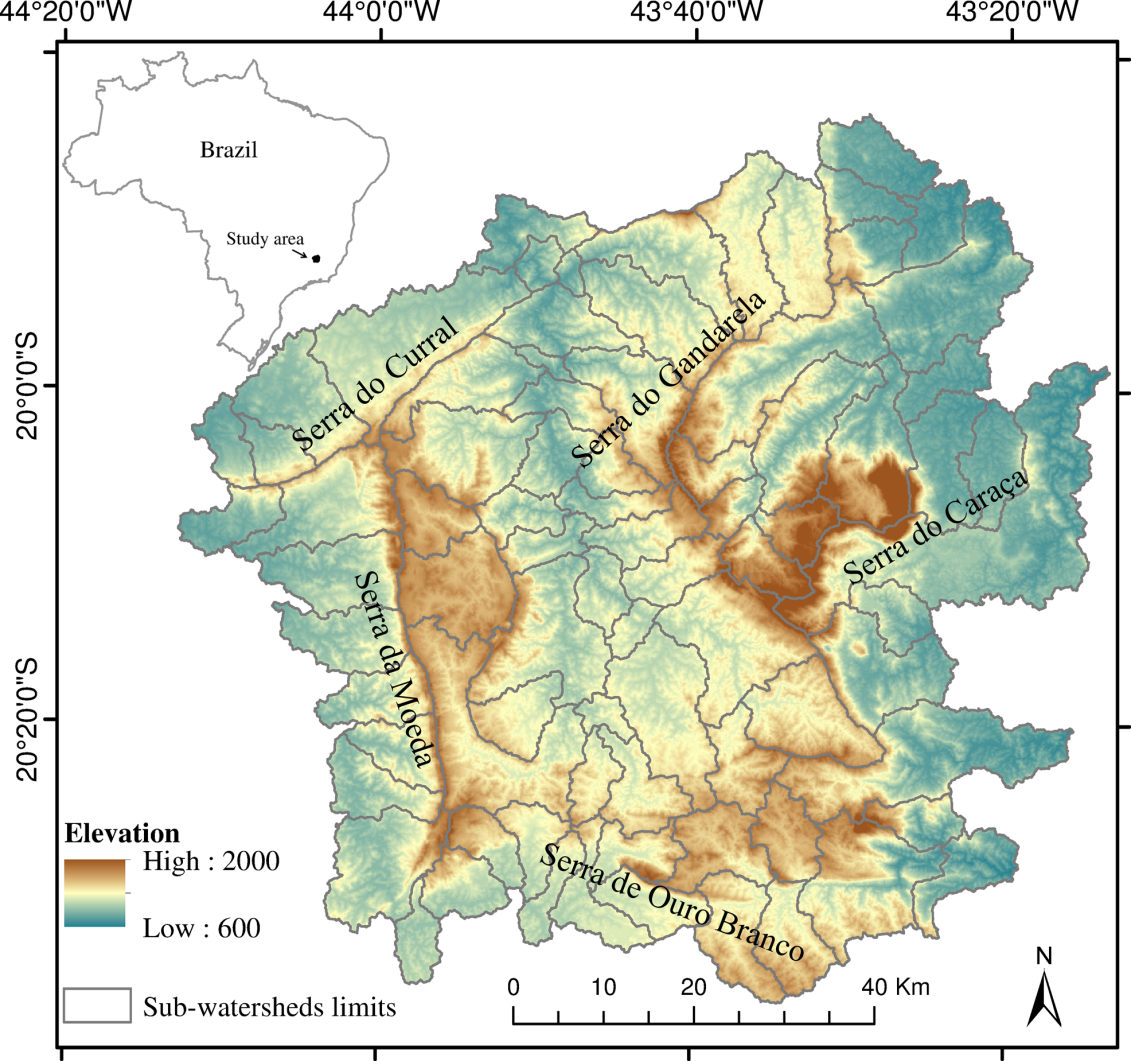
Map of the study area. Map representing the Iron Quadrangle’s selected sub-watersheds, the digital elevation model with its altitude range and the main ridges. The inset illustrates the location of the study area within Brazil and Minas Gerais state.

The Iron Quadrangle is about 7000 km^2^ and is located in the southeast of Brazil (Fig 1). Its name is due to the format of the alignments that delimit the region: Serra do Curral to the north, Serra de Ouro Branco to the south, Serra da Moeda to the west, Serra do Gandarela and Serra do Caraça to the east. The Iron Quadrangle is responsible for approximately 67% of Brazil’s measured iron ore production [27] and is submitted to increasing global demand for iron and steel [30]. The Iron Quadrangle is located within two of Brazil’s major watersheds, Rio Doce and Rio São Francisco. A subtropical latitude climate prevails, characterized by a dry winter and rainy summer, where places with higher rainfall indices have an annual mean of almost 2000 mm, and those with lower rainfall indices have an annual mean of 1400 mm [31]. The altitude ranges from 586 to 2087 m.a.s.l. Many vegetation types occur in the Iron Quadrangle, varying from tropical semi deciduous forest to rupestrian grasslands, due to the high geodiversity, different soil types and altitudinal/climate gradients [32,33]. We chose the Iron Quadrangle as a study region due to its high levels of biodiversity and endemism, the occurrence of relatively large natural areas, the presence of relevant watersheds providing water for one of the largest urban centers in Brazil, and due to the increasing anthropogenic pressures associated mostly with mining activities and urban expansion [29,30].

For the study area delimitation, we used the digital elevation model (DEM) available for the region, obtained from the “Advanced Spaceborne Thermal Emission and Reflection Radiometer” (ASTER GDEM). Using digital and automatic processing, we delimited sub-watersheds in the region with the GRASS GIS software [34]. Each sub-watershed had a minimum area of 36 km^2^. During this digital processing, we generated data on flow direction, flow accumulation and the definition of the drainage network (for more details see [35]). We then selected 80 sub-watersheds, using the criterion of intersecting the Iron Quadrangle alignments, the availability of maps and other necessary information for subsequent analysis. The total area summed approximately 6500 km^2^ (Fig 1).

### Modeling key ecosystem services

#### The InVEST model as baseline

For mapping and quantifying habitat quality and ecosystem services, we used InVEST (Integrated Valuation of Ecosystem Services and Tradeoffs), a GIS tool developed by Stanford and Minnesota Universities, World Wildlife Fund and The Nature Conservancy [36]. This geospatial tool helps to evaluate land use change impact on ecosystem services [37,38]. As cited by Nelson *et al*. [39] “InVEST is a suite of service models that use production functions to convert maps of land use and land cover (LULC), land management, and biophysical conditions into maps of service supply”. Therefore, the software has a generalization characteristic that is important for covering different landscapes, situations and needs. However, due to this characteristic, the InVEST software did not fulfill all our needs to model the ecosystem services of interest (see below). Thus, we developed a set of GIS tools to complete the tasks, which we described in the following sections. We selected the services to model based on the availability of data to determine them, and because they are the most used in ecosystem services payment projects [16].

#### Land use and land cover map

To obtain a LULC map for the study area that would serve as an input for all models, we mosaicked and edited maps provided by Vale S.A. company. These maps were developed in 2008 [40]. Using ArcGIS 10.2 software [41] for visual analysis, we chose the maps that were most consistent for each class in each sub-watershed, having as reference LandSat 8 OLI images from 2013, obtained from *United States Geological Survey* website (USGS), and RapidEye images from 2009, provided by Minas Gerais Institute of Forestry (IEF-MG). We combined different spectral bands and produced several image compositions to facilitate visual identification of LULC classes (see the definition of classes in Table A in the S1 Appendix). In this step, we took the 2013 LandSat 8 images as reference and examined them for LULC changes that had occurred since 2008, the year that the Vale S.A. company maps refer to. Therefore, we fixed a 1:20,000 scale, performed a new visual analysis using manual edition and generated a more accurate LULC map. After all these steps, we have created a new LULC map for the year 2013, independent of the initial ones, which we used in all subsequent analyses.

We validated the LULC map through 471 ground truth points collected throughout the entire region in 2014. Field routes were predetermined, aiming to cover the majority of sub-watersheds. In order to quantify the classification’s accuracy, we generated a confusion matrix using the cover classes from our map and from the ground truth points and calculated omission and commission errors. We aimed to have overall classification accuracy greater than 80%. Table B in the S1 Appendix presents the results of this matrix.

#### Habitat quality

Biodiversity *per se* is not considered an ecosystem service, but it is well-recognized as being important to ecosystem processes and to the maintenance of several ecological functions and services (e.g. primary production, disease and pest control) [2,42]. We had the same assumptions as the InVEST models [36]: i) a positive relationship exists between habitat quality and biodiversity; ii) habitat quality is a proxy for quantity and quality of available resources; iii) habitat quality decreases with the proximity of anthropogenic land use, but the intensity of this decrease varies according to the land use class. The first necessary inputs for the InVEST model were a habitat and threat raster. We defined LULC classes using a binary system in which zero corresponded to a threat LULC class and one corresponded to a habitat LULC classes. In addition, the InVEST model considers the distance between the threat’s source and the habitat. The intensity of impact on habitat quality caused by a specific threat decreases with distance according to a decaying exponential function (see Eq 3).

In our study, we were not interest in punctual impacts, but in the impacts that percolate the landscape and affect biodiversity (*e*.*g*. noise and air pollution, reduction of water quality and quantity downstream, landscape fragmentation and habitat edges effects). As the Iron Quadrangle has a relatively high altitudinal range, we can see its hills and mountains as geomorfometric barriers to the impacts caused by some LULC threats (S1 Fig). We adapted the InVEST model to incorporate hilly conditions as a barrier to threat propagation. As neither InVEST nor ArcGIS currently allow this function, we coded the supplementary procedures within GRASS GIS. First, we used the slope (in degrees, derived from a digital elevation model with 30-m spatial resolution) and its cosine to correct the impact distance from the threats (d_a_), accounting for the ups and downs of relief:

$$d_{a}=30/\;\text{cos}\;\theta$$ (1)

where the number 30 is due to the raster resolution, and θ is the slope in degrees (see S1 Fig). Then, we did the maximum relief curvature analysis in GRASS GIS to identify the position where the relief became a barrier to threat propagation. To calculate the maximum relief curvature, we inputted a 500-m radius of influence around every pixel and considered the 20% highest curvatures as a barrier to threat propagation. We put a very high value (in this case equal to 300) on these top 20% pixels to impose a geomorfometric barrier, in which the distances following it would be too great for any impact to be significant. We summed the maximum curvature and the “d_a_” raster to obtain a “distance cost surface” for the subsequent analysis, *i*.*e*., we created a new relief distance raster that attenuated the impact factor of threats where a relief barrier exists. Lastly, with this new raster, we did a cost distance model in ArcGis software, treating each one of the threats as a source. With this, we obtained a “cost-relief-distance” (d_c_) for each pixel. To summarize, “d_c_” is equal to the cost distance model (CDM; measured in meters) of the sum of “d_a_” and the maximum curvature raster (MCR):

$$d_{c}=CDM\left(d_{a}+MCR\right)$$ (2)

Considering the LULC raster map of the study area, the impact (i_rxy_) of a pixel (y) with a certain threat class (r) over a habitat pixel (x) is equal to:

$$i_{rxy}=\text{exp}\left(-\left(\frac{2.99}{dmax}\right)d_{c}\right)$$ (3)

where “d_c_” is the obtained cost-relief-distance (in meters) between the pixels, and “dmax” is the maximum influence distance (in meters). To address this maximum distance, we consulted with 16 specialists that had knowledge about the study area and about different organism groups (mammals, birds, amphibians, reptiles and plants). We asked them to evaluate the maximum influence distances of all LULC threat classes (see Table C in the S1 Appendix), *i*.*e*., the maximum distance that a threat affect the quality of a habitat for the organisms groups. Landscape ecologists have increasingly used the expert knowledge information in their analyses [43–45], particularly when empirical data are not available for all set of species. We used the *Delphi* method [46], in which we send the questionnaire with a text description of the survey to experts via e-mail. Based on their responses, we calculated the average maximum influence distance. The experts received the summary of the initial results with a request to review their initial position. They could maintain the first answer if they were sure of it, or they were free to change their opinion. Based on the revised information, we calculated the median values of all distances obtained per threat. In addition, InVEST model also account for a weights raster (w_r_) of the LULC classes according to their habitat quality degradation capacity (see Eq 4). We obtained the weights values from the same expert knowledge technique, asking the specialists about the relative intensity of degradation of a LULC threat on the quality of a habitat on an organism (see results in Table C in the S1 Appendix).

LULC threats have little impact on protected areas, which usually have some kind of management project and administration policy for their protection. Brazil’s National System of Conservation Units (SNUC) provides categories for protected areas established in the country’s territory [47], which can be divided into strictly protected areas (IUCN Categories I-III) and sustainable use protected areas (IUCN Categories IV-VI). The InVEST model has an accessibility factor (β_x_) that reduces the impact of threats inside protected areas by a user define factor. We chose a value of 0.5 for β_x_ only for strictly protected areas. This factor reduced in half the impact of external threats inside these areas. We chose this value due to the effect of known management projects, administration policy and based in our expert judgment. We omitted one of the strictly protected areas from this evaluation, as it is a very recently created national park (Gandarela National Park, est. October 2014) and lacks a management project and administration policy. Moreover, we defined all evaluated habitats LULC classes as equally susceptible to all sources of threats.

Thus, the total level of threat (D_xj_) in a particular pixel (x) with a given habitat class (j) is given by the equation:

$$D_{xj}=\sum_{r=1}^{R}\sum_{y=1}^{Yr}(\frac{Wr}{\sum_{r=1}^{R}Wr})r_{y}i_{rxy}\beta_{x}$$ (4)

where r is a LULC threat, with r = 1, 2,…, R indexes all of the modeled degradation sources; y indexes all of the grid cells on r’s raster map; Yr indicates the set of grid cells on r’s raster map; i_rxy_ the result of Eq 3;w_r_ is the weights parameter; and β_x_ is the accessibility factor, both described above. The habitat quality (Q_xj_) in a given pixel (x) is the result of the equation:

$$Q_{xj}=1-\left(\frac{D_{xj}}{D_{xj}+0.5}\right)$$ (5)

#### Carbon stock

The natural stock of carbon benefits humans by acting as a climate regulator. In this study, we modeled carbon storage according to the amount of four main reservoirs: i) the aboveground living plant biomass; ii) belowground biomass, which includes the roots of these plants; iii) soil organic components, which represent the largest terrestrial carbon reservoir [48]; iv) dead organic matter present in the litter. We used data averages available in the literature for each LULC class (Table D in the S1 Appendix). All values were in Mg ha^-1^. We sought data that were from the same watersheds or had vegetation, climate and soil features similar to ones in the study area. We considered only terrestrial environments. For the forest class, it is described in the literature that edge effects reduce the aboveground and belowground biomass by increasing tree mortality in the first 100 meters [49–52]. We went beyond the InVEST model and took forest edge effects into consideration by reducing the biomass on the edge areas, and therefore reducing also the carbon stock ([52], and see S1 Appendix for the values of carbon stock used). In addition, the belowground biomass (BGB) of forest and urban classes were calculated according to the equation described by Pearson *et al*. [53]:

BGB=exp(1.0587+0.8836×lnAGB) (6)

where AGB is the aboveground biomass. We assumed that 50% of the stock biomass is carbon [54].

#### Sediment retention

The natural control of excessive erosion can benefit humans by, for example, increasing agricultural productivity, reducing flooding and pollutant transport, improving water quality, reducing sediment removal in reservoirs and improving the habitat quality for aquatic species. Therefore, it is directly related to water services, and can be used in ecosystem services payment projects. Sediment retention was estimated using the universal soil loss equation (USLE) [55], which consider LULC information along soil properties, rainfall data and elevation. Thus, the annual soil loss due to water runoff (A), measured in ton/ha/year, is the result of the equation:

A=R×K×LS×C×P (7)

where R is the rainfall erosivity (MJ/ha/(mm/h)); K (ton/MJ/ha/(mm/h)) is the soil erodibility factor; LS is the slope length-gradient factor; C is the cover-management factor (accounts for the ratio of soil loss in the specified crop/vegetation and management relative to continuously fallow and tilled land); and P is the support practice factor (represents the ratio of soil loss by a support practice to that of straight-row farming). The last three factors are dimensionless.

The rainfall erosivity index was calculated using the program NetErosividade [56]. The program allows to calculate the annual erosivity for any location in the state of Minas Gerais from data interpolation performed using neural networks. We chose the method proposed by Foster *et al*. [57] to calculate the kinetic energy and the erosivity index EI_30_. The map had a coarse resolution (900 m) but it was the only available map covering the whole region. In our case, we considered that precipitation rates did not vary significantly on a finer spatial scale than the one obtained, but did so on a temporal scale during one year [31]. We obtained the soil erodibility rate (which indicates the susceptibility of soil particles to be detached and carried by the rain) from studies in the literature for each soil type found in the region. The soil type map, provided by Vale S.A., had a 1:50,000 scale [40]. We also took the values for cover-management and support practice factors from the literature (see Tables E and F in the S1 Appendix), considering areas with similar characteristics; we previously observed these practices in the field. We obtained the LS factor from the digital elevation models cited for the delimitation of the study area. As the vegetation also retains eroded upstream sediment, the model also predicts a value for filtering sediments [36]. This field corresponds to the capacity of each LULC class to retain sediment coming from above the terrain and should be understood as a relative value (one class can retain more sediments compared to another one). We chose the values according to the relative density of vegetation found in each LULC class.

### Data analysis

We performed three steps to analyze the models’ outputs: we 1) verified the parameters that most influenced each model; 2) checked for the overlap between the models’ output; and 3) created a prioritization method for those areas that overlapped. We made these analyses in ArcGIS and R software (R Core Team, 2013). For the first step, we performed a sensitivity analysis to quantify how the spatial variance of model parameters influences the ecosystem service maps [58]. This was made through the standardized regression coefficient (SRC) analysis, which estimates the average, standard errors and 95% confidence interval of the relative contribution of each explanatory variable on each response variable: habitat quality, carbon stock and sediment retention [58]. The SRC varies between −1 and +1, with values near zero representing variables with low or null influence in the response variable (*i*.*e*. ecosystem service maps). We used the impact of each LULC threat and the accessibility factor as the explanatory variables for the habitat quality model. We used each one of the carbon pools as explanatory variables for the carbon stock model. For the sediment retention model, we used the USLE parameters and the sediment filtration factor as explanatory variables. To prepare a table with a response (ecosystem services) and explanatory variable (model parameters), we randomly selected 10,000 pixels of our entire region for analysis. Therefore, although we used some fixed parameters on the calculations of some explanatory variables, their values vary throughout space, which made this analysis possible.

As each service uses a specific unit of measure, they were not directly comparable. In the second step, we rescaled the three service maps from zero to 100, following the formula:

$$Z_{i}=\frac{X_{ij}}{Xmax}\times 100$$ (8)

where “X_ij_” is the value for ecosystem service “i” in pixels “j”; “Xmax” is the maximum score for ecosystem service “i” across all pixels; and “Zi” is the new score for that pixel. We previously log-transform the data only for the sediment retention results, because the amplitude of result values was too high. To assess the spatial correlation between the model results, we calculated Pearson’s correlation coefficients for each pair of services. We then assessed the ability to bundle the results of each model: Following Wendland *et al*. [15], we summed the areas containing an overlap of pixels with more than 0% of the highest value of each service, 15% or more, 30% or more, and so forth up to 90% or more.

For the third step, we selected to overlap the 20% of pixels that had the biggest scores (on the 0 to 100 range) of each model’s output. Next, we took only pixels where the overlap of at least two models occurred. The issue was that we had regions where only few pixels fall and we had to prioritize areas with a larger number of pixels.

In our study area, the habitat fragmentation is high and there are few natural patches with an area large enough to maintain high biodiversity for long term. Consequentially, the connectivity is important in any conservation spatial plan for the region. In these sense, we used functional connectivity-based approach to create a spatial aggregation index for each one of the pixels that overlapped in the third step. Functional connectivity is the capability of landscape facilitate organism movements among resource patches [59]. Thus, we used a 250-m radius focal statistical analysis, with the selected overlapped pixels in the third step as input [60]. We gave to each one of these overlapped pixels a core equivalent to the number of neighboring pixels within this radius. We chose this radius-distance due to the ocelot (*Leopardus pardalis*) movement capacity in an inhospitable landscape matrix, the ocelot being a felid species that is sensitive to the habitat loss occurring in our study region [60,61]. This species represents an approximation of the connectivity demands of vertebrate species that are sensitive to fragmentation and are forest dependent. Therefore, we obtained a priority gradient, ranging from low priority (pixels with low aggregation/connectivity) to high priority (pixels with high aggregation/connectivity). We can use this gradient as an initial step for conservation and for future projects of payments for environmental services.

## Results

The LULC map had a global accuracy of 82% (S2 Fig and Table B in the S1 Appendix). We excluded from this analysis the LULC classes with less than 2% of the total study area (Table 1). We found that habitat classes (Cerrado, forest, rupestrian grasslands, and water bodies) accounted for 70% of the study area. Nevertheless, many natural habitats are suffering direct pressure from human-disturbed LULC classes. For instance, our study area has more forest edge area than it has interior forests.

**Table 1:** Area of the each land use and land cover (LULC) classes within Iron Quadrangle, Minas Gerais, Brazil.

**LULC name**	**Area (Km**^**2**^**)**	**Percent of the total area**
Agricultures	13.44	0.21
Cerrados	962.96	14.83
Eucalyptus plantations	316.56	4.88
Forest (interiors)	1,249.63	19.24
Forest (edges)	1,637.97	25.23
Mining areas	189.80	2.92
Pastures	893.93	13.77
Roads network	120.44	1.85
Rupestrian grasslands	623.87	9.60
Urban areas	436.85	6.73
Water bodies	47.91	0.74
TOTAL	6493.36	100

In this table, we considered forest and forest edges separately.

**Fig 2:**
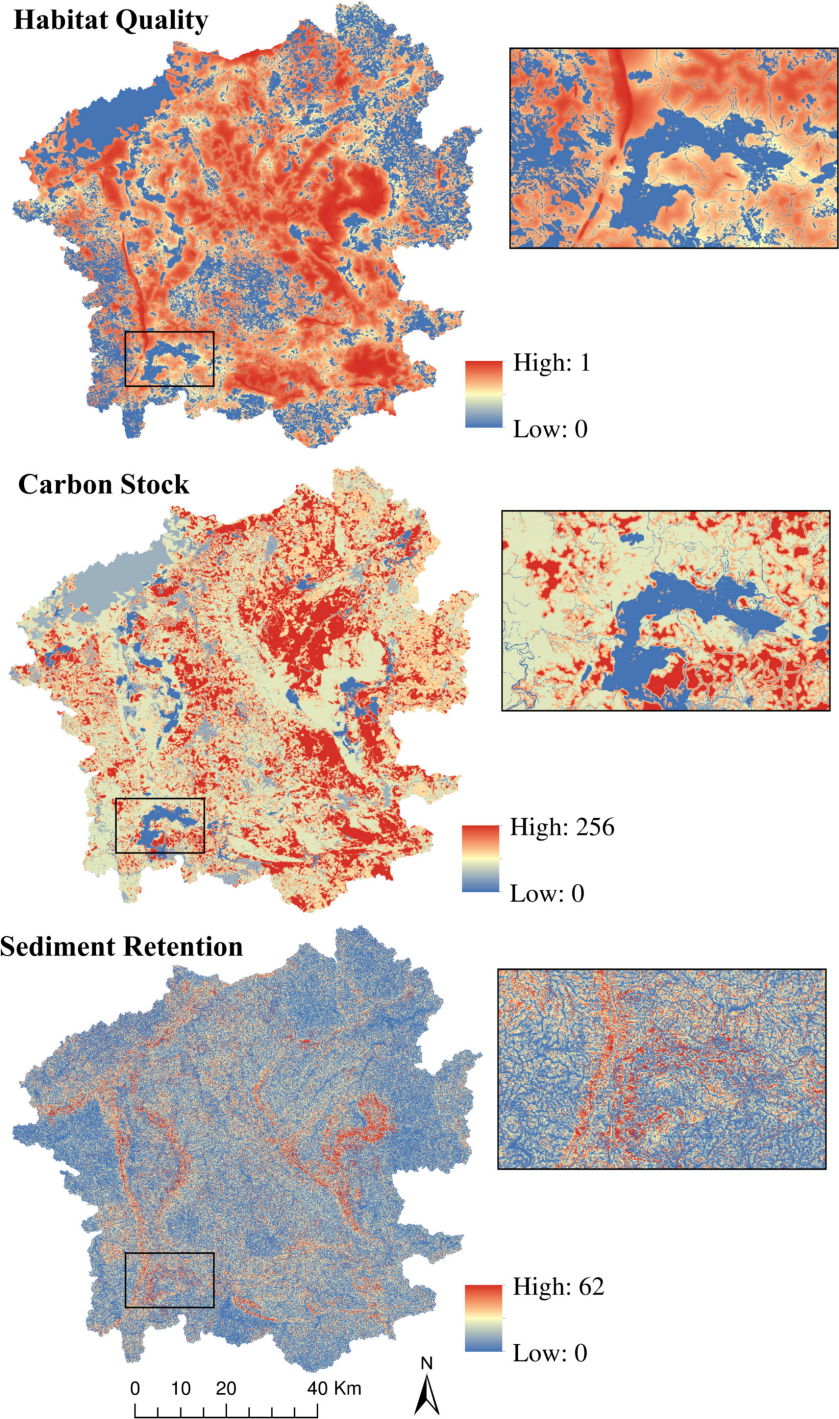
Ecosystem services resultant maps. Iron Quadrangle’s output maps and their quantitative variation for each one of the models: habitat quality at the top, carbon stock (tons of carbon/ha) in the center and sediment retention (tons/ha/year) below. The three insets show the same zoomed area for its respective model.

The three ecosystem services presented great variation in the Iron Quadrangle (Fig 2). The habitat quality model ranged from zero to 0.99 (mean = 0.52; standard deviation ±0.35). Despite the high anthropogenic impact of many areas devoted to eucalyptus, pastures and mining activities, there are some places with relatively high habitat quality that promote appropriate conditions for sensitive species like the ocelot *Leopardus pardalis* [60]. Carbon stock varied from zero to 255.8 tons/ha (144.6 ±69.22). The log_10_ of the sediment retention model had values between 0 and 61.7 tons/ha/year (6.7 ±7), meaning that sediment retention was a diffuse ecosystem service, with few areas providing very high rates of retention, and many areas providing medium to low rates.

### Sensitivity analysis

**Fig 3:**
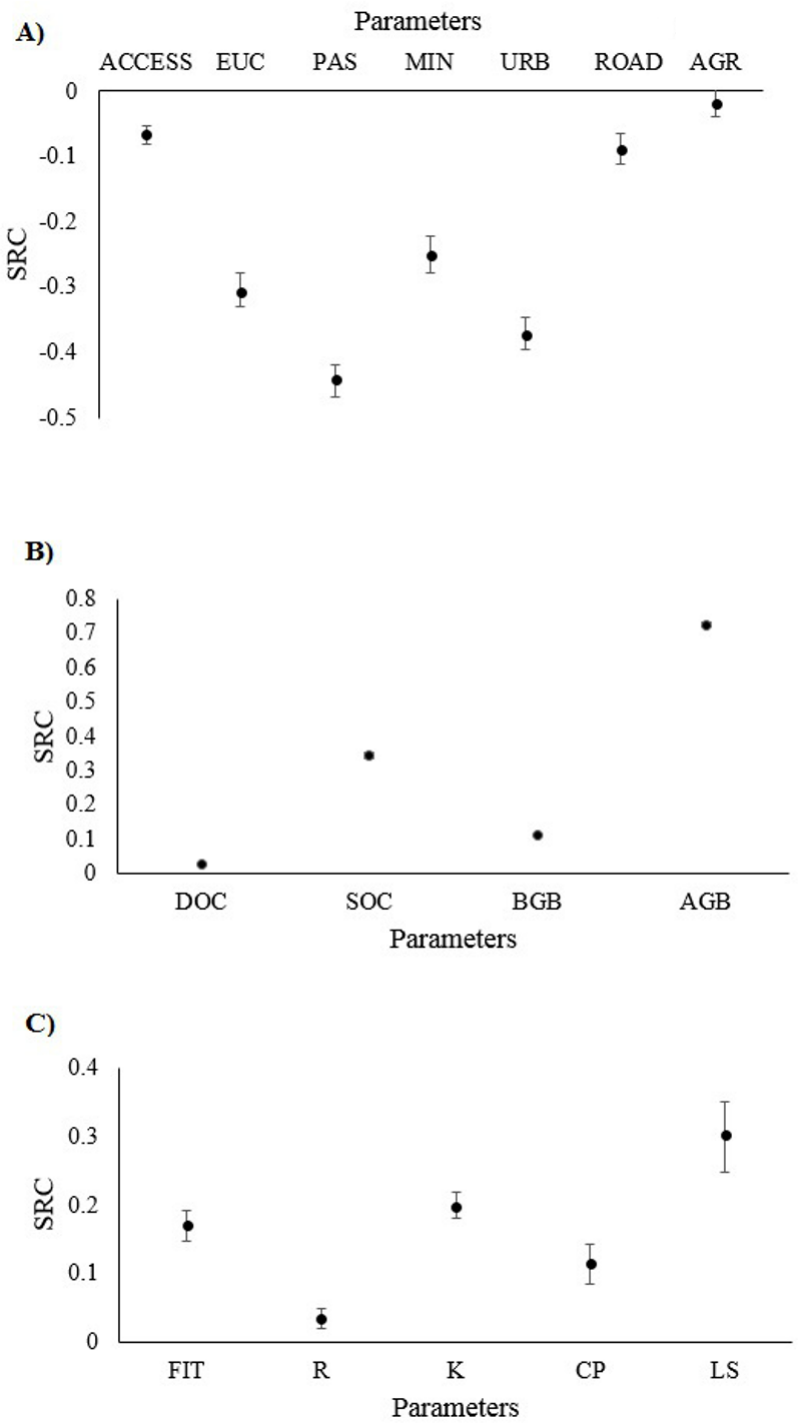
Sensitivity analyses of the parameters used in three models, measuring their influences on output ecosystem services maps. (A) Standardized regression coefficient (SRC) for the habitat quality model, where ACCESS is the accessibility parameter of anthropogenic land use land cover class impacts in conservation units, and the others are impacts caused by eucalyptus plantations (EUC), pasture (PAS), mining areas (MIN), urban areas (URB), the roads network (ROAD), and agriculture fields (AGR). (B) The SRC values for the carbon stock model, where DOC is the dead organic carbon, SOC is the soil organic carbon and BGB e AGB correspond to the carbon stock in below- and aboveground biomass. (C) The SRC values for the sediment retention model, where FILT is the sediment filtration parameter and R, K, C, P and LS are the USLE factors.

The pasture and urban areas classes were the main factors influencing the habitat quality (SRC = −0.44 ±0.01 and SRC = −0.37 ±0.01, respectively), both with strong negative influence (Fig 3A). This could be because these threat classes are spread throughout the entire landscape, affecting almost all habitat fragments. In addition, the sensitivity analysis showed that the accessibility layer had low or null influence (SRC = −0.06 ±0.01), mostly due to the low impact of threat LULC classes on the strictly protected areas in the study region. For the carbon stock model, the aboveground stock (SRC = 0.72 ±0.01) had the strongest influence in the model results’ variation (Fig 3B), mostly because of the great differences in natural vegetation types in the region, ranging from grass lands (low aboveground biomass) to forests (high aboveground biomass). The results for the sediment retention model (Fig 3C) showed a stronger influence of the LS factor (SRC = 0.3 ±0.02), and intermediate influence of the K factor (SRC = 0.2 ±0.01) and sediment filtration (SRC = 0.17 ±0.01).

### Synergism and conservation priorities

**Fig 4:**
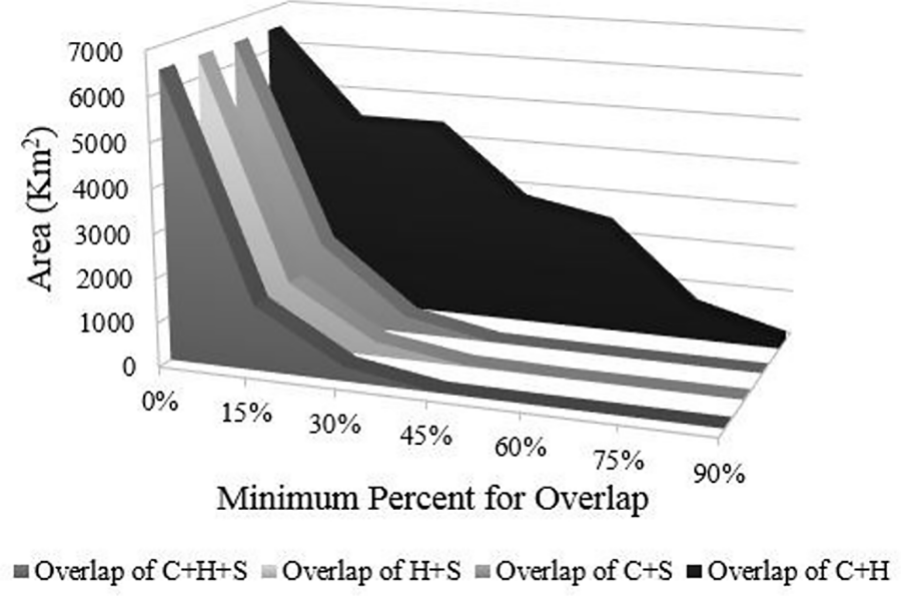
Sum of the total area that overlapped when the services are bundled together in each one of the minimum percentiles. C is the carbon stock service, S the sediment retention and H the habitat quality.

The modeled services and habitat quality had different spatial distributions, as shown by the correlation coefficients in Table 2, although we found some areas with either a high or a low value of multiple services. Bundling the services and habitat quality, we found weak spatial overlap among all three after a value of 15% or more of each model output (Fig 4). This overlap reached zero nearly 45% or more of each service. Considering the overlap between sediment retention and the other two models separately, we found the same pattern. This is mostly because the sediment retention model had the majority of pixels with intermediate values spread across the landscape. Notwithstanding, there was strong spatial overlap of up to 60% for habitat quality and carbon, showing a higher synergism of those two models’ results. This is because the remaining forest fragments are large enough to have forest blocks that do not suffer from edge effects and are located in regions that are topographically protected from the impacts of anthropogenic land uses.

**Table 2:** Correlation Coefficients for each pair of models.

**Correlation Coefficients**	**Habitat Quality**	**Sediment Retention**
Carbon Stock	0.55	−0.07
Habitat Quality	1	0.10

We obtained the coefficients through Pearson’s correlation.

**Fig 5:**
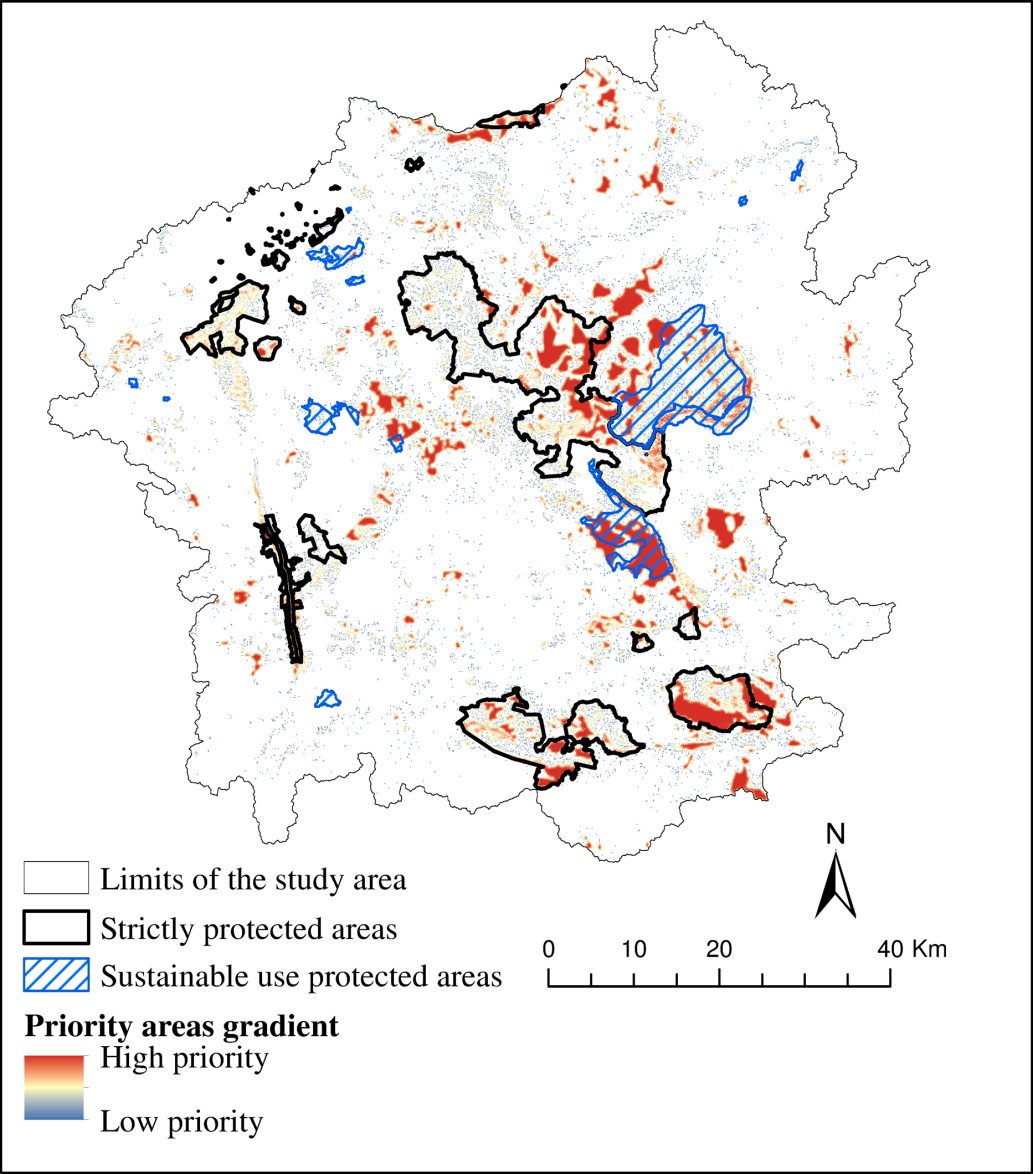
Gradient of priority areas and the conservation units present in the study region. Pixels nearer the red color have a high aggregation index, and the ones near the blue color have a low value for the same index.

We produced a map that decision makers could use as a source of information for determine priority areas for conservation (Fig 5). These areas correspond to 13% (826 km^2^) of the study region. About 30% of these priority areas are already in strictly protected areas (counting the recently created Gandarela National Park), and 12.2% are in sustainable use protected areas. As there are many kinds of sustainable use protected areas in Brazil, this study only considered the ones that assure a minimum biological conservation status [47], as do the private reserves of natural heritage (RPPN in Portuguese acronym) and the National/State Forest (FLONA/FLOE). Also of interest in Fig 5 is the presence of priority areas with high connectivity that are not within any existing protected areas.

## Discussion

Our efforts have produced a first step for planning conservation priority areas in the Iron Quadrangle. The outputs that are generated using the approach we propose bring valuable information for regional planning, and this have a great potential to reduce conflicts between socioeconomic and conservation interests. The method proposed here presents a promising alternative to find the synergism between ecosystem services and biodiversity protection. It provides an opportunity to consider ecosystem services as a new argument for supporting decision making in a conservation framework, while simultaneously incorporating human needs and demands into the priority areas planning process [3,21]. Our study demonstrated that, even with limited information available, we could quantitatively access and analyze areas with a high capacity for providing ecosystem services throughout the space. Following the transparent and highly replicable method described in this study, conservation planners can better determine the areas within the landscape that provide multiple goals and trade-offs. The ecosystem services approach is increasingly necessary, as the human population and economic activities continue to grow [3].

One key aspect of this process was to determine how ecosystem services and biodiversity could be bundled together. We found low congruence between sediment retention and the other two models’ results—carbon stock and habitat quality. As the Iron Quadrangle has a very rugged topography and this model is sensitive to the LS factor, we argue that it could have reduced the spatial correlation with the other two models. Despite this fact, the sediment retention service is important because it highlights areas where landowners need to preserve riparian vegetation and the tops of hillsides, particularly under rugged terrain conditions. The Brazilian National Forest Code already determines the sizes for riparian forest buffers to be preserved by landowners [62], but this is not always accomplished [63]. Thus, environmental liabilities have a negative impact on sediment retention services.

The correlation of terrestrial carbon and habitat quality is still controversial, having different patterns in different scales and landscapes [26,64–67]. In this study, carbon stock had a high congruence with habitat quality when compared to sediment retention. The maintenance of those congruent areas could be a target for economic incentives, such as the Warsaw Framework for Reducing Emissions from Deforestation and Forest Degradation, known as REDD+ [68]. For this, the government and landowners have to demonstrate emission reductions through improved carbon stocks, forest protection and/or sustainable management, in comparison to a “business-as-usual” scenario.

Notably, the relief-factor that we applied to the habitat quality model reduced the impact of anthropogenic areas in the habitats LULC classes. In areas of very rugged topography such as the Iron Quadrangle, we have to consider geomorfometric barriers for these impacts and for species movement and we must think beyond the linear distances. We argue that we have taken an important step towards the improvement of the InVEST habitat quality model, which has received only a few updates of early versions ([69] compared to [36]). For this, we merely added the digital elevation model as an input, maintaining the simplicity and replicability of the model, since this information is readily available and already used in other InVEST models.

Among the priority areas found in our analysis, 42.2% overlapped with protected areas. The ones that overlapped with strictly protect areas could receive financial support through payments for those ecosystem services. These reserves and parks usually lack financial support and management projects [6], do not always restrict nearby deforestation [70], and suffer pressures from local community because they have high opportunity cost [10,12,71,72]. Ecosystem services are already helping to solve those problems in some parts of the world [13,72–74] and can assist in this case. In private reserves, landowners could earn additional income based on the valuation of social benefits derived from ecosystem services, incorporating the positive externalities into the value of their protected areas for sustainable use. For areas that did not overlap with any conservation units, yet have high aggregation indices, we recommend the implementation of strictly protected natural reserves (IUCN Category Ia). Those areas are extremely important for maintaining landscape connectivity and are large enough to conserve high rates of ecosystem services, permitting many sensitive species to persist. The areas around them and around those with lower connectivity indices could be sustainable use protected areas, given adequate management aimed at future ecosystem services provision. Together, our priorities areas and the conservation units of the Iron Quadrangle cover 20% of the study region, exceeding that of the eleventh terrestrial environment target established by the Aichi Biodiversity Targets for 2020 [5].

We considered ecosystem analysis very important in the Iron Quadrangle region because of increasing mining pressures [30] that could generate high social and economic externalities in the region. The productive mining sector is expanding in the study area and creating new open pit mines, leading to losses in vegetation and soil carbon stocks as well as more erosion siltation of rivers and losses in groundwater recharge, triggering problems for populations downstream [75,76]. This is worrying if we consider that 43% of water consumption for the state’s capital metropolitan area depends on the flow of rivers in the region [77]. This economic activity threats an ecosystem with very high biodiversity and with many endemic species, the rupestrian grasslands, a particular ecosystem found in areas with high altitude [29]. According to your expert knowledge research (see Table C in the S1 Appendix), mining areas have intense and long-distance impact. Therefore, if this activity rapid expand, there will be great loss in ecosystem services and biodiversity.

Quantifying other services in the landscape is necessary to understand the opportunities for financial and social support for conservation. Services that provide direct benefits, such as timber production or food provision, can have many trade-offs for biodiversity conservation [3]. To model other hydrological services as water retention, clean water production and groundwater recharge would be beneficial for a conservation plan process. We could not done this in this work due to lack of regional data availability. There is a need to model the presence of water bodies as well.

We need a future research that uses more robust methods as spatial optimization techniques (*e*.*g*. [12,20]) and a more direct biodiversity index (*e*.*g*. [15,65]). In addition, there were some limitations in our models. The InVEST models used here do not account for variability in carbon stock and sediment retention within specific LULC and soils types. We only account the variation in forest edges. In addition, we based the habitat quality model in expert knowledge, and considered a new approach with geomorfometric barriers that may reduce impacts of threats. In this sense, there is a need that researchers should validate the models to ensure their efficacy. All methods used and proposed in the study, will depend on data availability for the region of interest (some of our inputs were from secondary data). By sure the use of empirical data about how landscape structure and land cover influences the species could improve the models. Besides, the functional connectivity approach used could have its priority results changed if someone is interested in different species or a group of species. For example, if the function distance was smaller than the one we chose, *i*.*e*. choose a more sensitive species to fragmentation, there will be less high-priority areas, as the search distance for aggregate/connected pixels will be smaller. If the distance were higher, the opposite will occur. In the first case, an example could be the critically endangered northern muriqui (*Brachyteles hypoxanthus*), a forest dependent primate which has an unconfirmed record in Iron Quadrangle [78]. An example of a less sensitive mammal species occurring in the Iron Quadrangle is the tapir (*Tapirs terrestris*), listed as Vulnerable by IUCN [78].

Moreover, we need to account for the additionality of our priority areas [15,18], because if they are going to be preserved or not deforested in the future, they do not need to be prioritized and the economic resources can be allocated to other places [17]. This could be done with projections of probable future scenarios that encompass stakeholders needs and deforestation rates [37,79–82]. Finally, it is also important to quantify and spatially analyze the demand for services [17]. In the case of the services described here, the scale and location of service provision do not equal the scale and location of its beneficiaries. Carbon has a local supply and global beneficiaries, and sediment retention has supply and demand in different spatial regions of the landscape. We believe that the spatial integration of biodiversity targets, ecosystem services provision and direct beneficiaries of pristine habitats could provide stronger arguments for conservation policies in conflict regions.

## Supporting Information

S1 Appendix Tables with data about the Land use land cover (LULC) class names and descriptions, confusion matrix and the input dataset for each model used in this work. (DOCX)

S1 Fig Representation of geomorfometric barriers reducing impacts from land use land cover threat classes and of correction of distance from the threats (d_a_). The gradient of colors in the arrows represent the impact reduction with the distance from its source (urban area or pastures). The slope in degrees was use to obtain the d_a_ distance, also reducing impact intensity in the natural land use land cover class (forest in this figure case). Design: Campestris.
(TIF)

S2 Fig Land use land cover map obtained for the Iron Quadrangle study region, showing each one of the classes found in the mapping process. (TIF)
