# Supplementary material for: Ecosystem Services Modeling as a Tool for Defining Priority Areas for Conservation
Source: PLoS One. 2016 May 4;11(5):e0154573. doi: 10.1371/journal.pone.0154573 (PMC4856429; doi:10.1371/journal.pone.0154573)
Supplement: S1 Appendix — (DOCX) [file pone.0154573.s001.docx]

**Supporting Information**

**Table A - Land use land cover (LULC) class names and descriptions found in the Iron Quadrangle region.**

| LULC class name | Description |
| --- | --- |
| Agricultural fields | Mosaic cropland (50-100%); vegetation (grassland/shrubland/forest) (0-50%) |
| Cerrado | Brazilian savanna vegetation/natural grasslands/shrubland |
| Eucalyptus plantations | Mosaic eucalyptus plantations (50-100%); vegetation (grassland/shrubland/forest) (0-50%) |
| Forest | Open-Closed (>40%) semi-deciduous Atlantic Forest |
| Mining areas | Areas with opencast mines. Includes buildings, associate industrial infrastructure, and small water bodies created by mining. |
| Pasture | Mosaic pasture (50-100%); vegetation (grassland/shrubland/forest) (0-50%) |
| Roads network | Highways and roads. Minimum width of 30 meters. |
| Rupestrian grasslands | Shrub and grasslands, typical from altitudes ranging from 900 to 2000 meters. |
| Urban areas | Mosaic of Buildings, roads and artificial surface areas (50-100%)/vegetation within urban areas (<50%) |
| Water bodies | Natural and anthropogenic water bodies |

Data used for mapping and as input in the three models – habitat quality, carbon stock and sediment retention.

**Table B - Confusion matrix for the land use land cover map.**

Using only the LULC class that had more than 2% of the total study area . The columns represent the number of ground truth points and the lines represent the pixels classification in this study. The commission and omission errrors are the proportion of the errors in the lines and columns respectively.

|  | Cerrado | Rupestrian grasslands | Eucalyptus | Forest | Mining areas | Pasture | Urban areas | *Total* | Commission Errors |
| --- | --- | --- | --- | --- | --- | --- | --- | --- | --- |
| Cerrado | 36 | 1 | 2 | 3 |  | 5 | 2 | *49* | 0.27 |
| Rupestrian grasslands | 6 | 47 | 2 | 2 | 1 | 1 |  | *59* | 0.20 |
| Eucalyptus | 1 |  | 38 | 2 | 1 | 2 |  | *44* | 0.14 |
| Forest | 6 | 6 | 9 | 118 |  | 10 | 2 | *151* | 0.22 |
| Mining areas | 3 | 1 |  |  | 17 |  |  | *21* | 0.19 |
| Pasture | 3 |  | 6 | 1 | 1 | 90 | 2 | *103* | 0.13 |
| Urban areas | 2 |  |  |  |  | 2 | 40 | *44* | 0.09 |
| *Total* | *57* | *55* | *57* | *126* | *20* | *110* | *46* | *471* |  |
| Omission Errors | 0.37 | 0.15 | 0.33 | 0.06 | 0.15 | 0.18 | 0.13 |  | 0.82 |

**Table C – Habitat quality model input parameters.**

| LULC Name ^a^ | Intensity | Maximum Distance (Km) |
| --- | --- | --- |
| Agricultural fields | 7.5 | 1 |
| Eucalyptus | 6.5 | 1 |
| Mining areas | 10 | 3 |
| Pastures | 7 | 1 |
| Roads network | 7 | 1 |
| Urban areas | 7.5 | 3 |

The intensity and maximum distance for each land use land cover class considered as threat; values obtained from specialist consultants (n=16).

^a^ Refer to S1 Table for LULC classes descriptions

**Table D – Inputs values used in the carbon stock model.**

| LULC Name^a^ | Aboveground Biomass | Belowground Biomass | Soil Organic Carbon (40 cm) | Dead Organic Carbon |
| --- | --- | --- | --- | --- |
|  | Mg ha^-1^ | | | |
| Agriculture fields | 7.2 | 1.9 | 62.44 | 1.1 |
| Cerrado | 2.7 | 15.088 | 90.684 | 0.96 |
| Eucalyptus | 56.7 | 9.9 | 74.3 | 7.4 |
| Forest | 134.0 | 27.6 | 90.6 | 3.6 |
| Forest edges | 69.0 | 13.2 | 90.6 | 3.6 |
| Mining areas | 0.0 | 0.0 | 0.0 | 0.0 |
| Pasture | 2.9 | 7.7 | 94.6 | 1.1 |
| Roads network | 0.0 | 0.0 | 0.0 | 0.0 |
| Rupestrian grasslands | 2.8 | 15.088 | 90.684 | 0.96 |
| Urban areas | 15.0 | 3.8 | 41.0 | 0.0 |
| Water bodies | 0.0 | 0.0 | 0.0 | 0.0 |

Data for soil organic carbon, dead organic carbon, aboveground biomass and belowground biomass carbon by land use land cover (LULC) class, obtained from literature^b^

^a^ Refer to S1 Table for LULC classes descriptions

^b^ References: [1-10].

**Table E – Mean values for K factor (erodibility) used in the universal soil loss equation (USLE) for each soil type, obtained from literature**^a^

| Soil type | K |
| --- | --- |
| Argisol | 0.04450 |
| Cambisol | 0.02314 |
| Gleysol | 0.03585 |
| Red Latosol | 0.00962 |
| Yellow-red Latosol | 0.01717 |
| Fluvic Neosol | 0.042 |
| Litholic Neosol | 0.045 |
| Quartzipsamment Neosol | 0.1448 |

^a^ References: [11-16]

**Table F - Sediment retention model input table**.

| LULC Name^a^ | C | P | Sediment Filtration (%) |
| --- | --- | --- | --- |
| Agricultural fields | 0.18 | 0.4 | 40 |
| Cerrado | 0.042 | 1 | 70 |
| Eucalyptus | 0.016 | 1 | 70 |
| Forest | 0.012 | 1 | 95 |
| Mining areas | 1 | 1 | 0 |
| Pasture | 0.052 | 1 | 50 |
| Roads network | 1 | 1 | 0 |
| Rupestrian grasslands | 0.042 | 1 | 60 |
| Urban areas | 0.1 | 1 | 3 |
| Water bodies | 0.01 | 1 | 10 |

For universal soil loss equation (USLE): cover and management factor (C), support practice factor (P) and sediment filtration factor by land use land cover (LULC) class, obtained from literature^b^

^a^Refer to Supp. Mat. S1 Table for LULC classes descriptions

^b^References: [17-19]

**References**

1. Castro EA de, Kauffman JB. Ecosystem structure in the Brazilian Cerrado: a vegetation gradient of aboveground biomass, root mass and consumption by fire. J Trop Ecol. 1998;14(3):263–83.

2. Cunha GDM, Gama-Rodrigues AC, Gama-Rodrigues EF, Velloso ACX. Biomass, carbon and nutrient pools in montane Atlantic forests in the north of Rio de Janeiro State, Brazil. Rev Bras Ciência do Solo. 2009;33:1175–85.

3. Gatto A, Barros NF De, Ferreira R, Ribeiro I, Leite HG, Leite FP, et al. Carbon storage in the soil and in the biomass of eucalypt plantations. Rev Bras Ciência do Solo. 2010;34:1069–79.

4. Hutyra LR, Yoon B, Alberti M. Terrestrial carbon stocks across a gradient of urbanization: a study of the Seattle, WA region. Glob Chang Biol. 2011;17:783–97.

5. Lilienfein J, Wilcke W. Element storage in native, agri-, and silvicultural ecosystems of the Brazilian savanna. Plant Soil. 2003;254:425–42.

6. Metzker TIF, Spósito TC, Martins MTF, Horta MB, Garcia QS. Forest dynamics and carbon stocks in Rio Doce State Park – an Atlantic rainforest hotspot. Curr Sci. 2011;100(12):2093–8.

7. Rangel OJP, Silva CA. Carbon and nitrogen storage and organic fractions in latosol submitted to different use and management systems. Rev Bras Ciência do Solo. 2007;31:1609–23.

8. Pearson T, Walker S, Brown S. Sourcebook for land use, land-use change and forestry projects. Washington, DC: BioCarbonFund; 2005. 62 p.

9. Pouyat R, Groffman P, Yesilonis I, Hernandez L. Soil carbon pools and fluxes in urban ecosystems. Environ Pollut. 2002;116:107–18.

10. Pulrolnik K, Barros NF De, Silva IR, Novais RF, Brandani CB. Carbon and nitrogen pools in soil organic matter under eucalypt, pasture and savanna vegetation in Brazil. Rev Bras Ciência do Solo. 2009;33:1125–36.

11. Beskow S, Mello CR, Norton LD, Curi N, Viola MR, Avanzi JC. Catena Soil erosion prediction in the Grande River Basin , Brazil using distributed modeling. Catena. Elsevier B.V.; 2009;79:49–59.

12. Bloise GDLF, Júnior OA de C, Reatto A, Guimarães RF, Martins E de S, Carvalho APF. Evaluation of natural susceptibility to erosion of the Olaria basin -DF. Planaltina, DF: Embrapa; 2001. 33 p.

13. Correchel V, Bacchi OOS, De Maria IC, Dechen SCF, Reichardt K. Erosion rates evaluated by the 137Cs technique and direct measurements on long-term runoff plots under tropical conditions. Soil Tillage Res. 2006;86(2):199–208.

14. Mannigel AR, Carvalho M de P, Moreti D, Luciano da RM. Erodibility factor and loss tolerance of soils of São Paulo state . Acta Sci. 2002;24(5):1335–40.

15. Silva AM da, Silva MLN, Curi N, Avanzi JC, Ferreira MM. Rainfall erosivity and erodibility of Cambisol and Latosol in the region of Lavras, south of Minas Gerais . Rev Bras Ciência do Solo. 2009;33(6):1811–20.

16. Silva MLN, Curi N, Oliveira MS, Ferreira MM, Neto FL. Comparison between direct and indirect methods for determination of erodibility in oxisols under Cerrado. Pesqui agropecuária Bras. 1994;29(11):1751–61.

17. D’Andréa AF, Silva MLN, Curi N, Guilherme LRG. Carbon and nitrogen stock and forms of mineral nitrogen in soil under different management systems. Pesqui agropecuária Bras. 2004;39(2):179–86.

18. Silva MA da. Modeling spatial of water erosion in the Doce River Valley, Central-East region of Minas Gerais state – Brazil. M.Sc. Thesis. Universidade Federal de Lavras; 2009. Available from: http://repositorio.ufla.br/handle/1/3582 Accessed 28 March 2014.

19. Yang D, Kanae S, Oki T, Koike T, Musiake K. Global potential soil erosion with reference to land use and climate changes. Hydrol Process. 2003;17:2913–28.
