# Supplementary figures and images for: Ecosystem Services Modeling as a Tool for Defining Priority Areas for Conservation
Source: PLoS One. 2016 May 4;11(5):e0154573. doi: 10.1371/journal.pone.0154573 (PMC4856429; doi:10.1371/journal.pone.0154573)

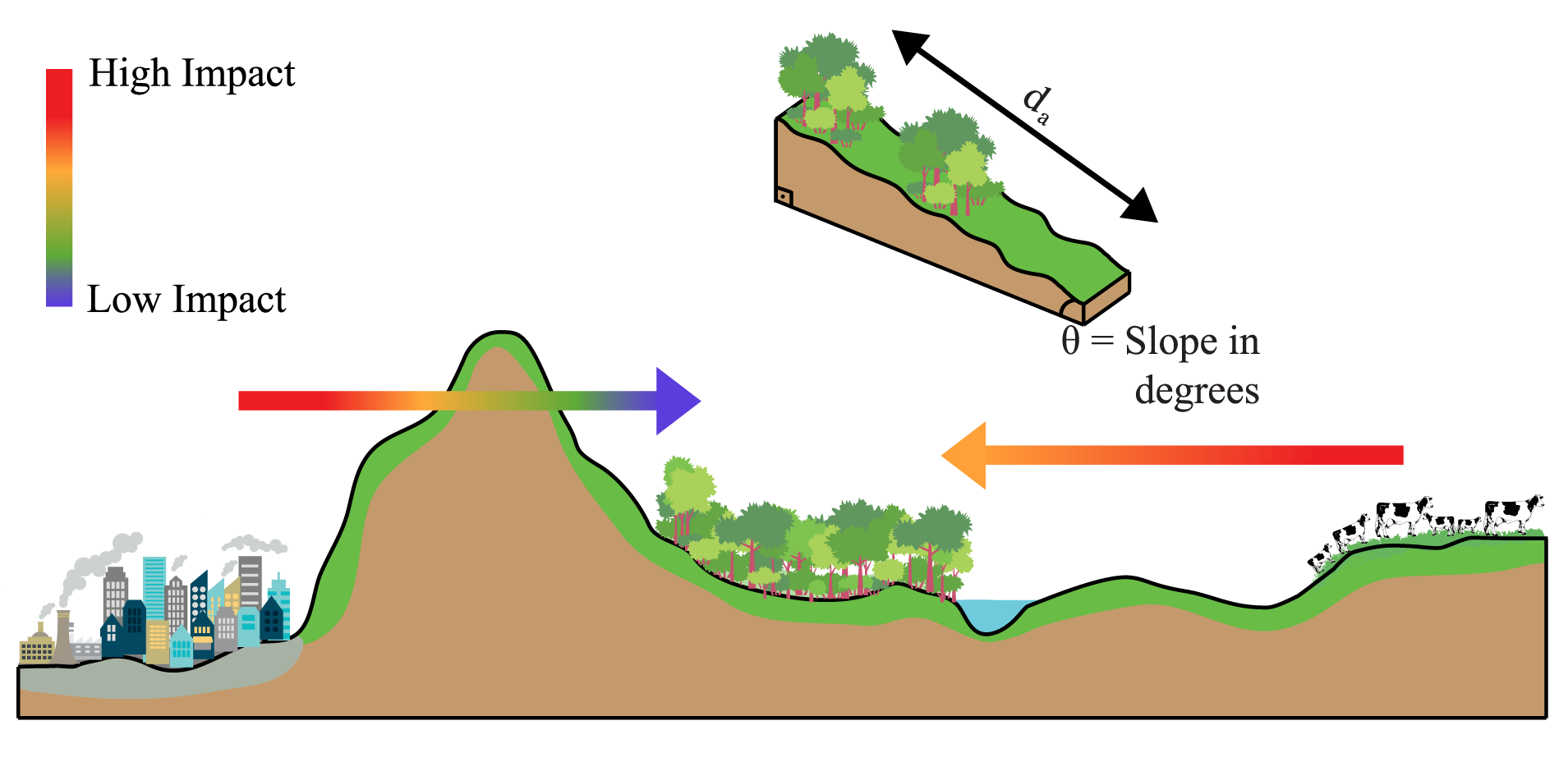

Supplement: S1 Fig — The gradient of colors in the arrows represent the impact reduction with the distance from its source (urban area or pastures). The slope in degrees was use to obtain the da distance, also reducing impact intensity in the natural land use land cover class (forest in this figure case). Design: Campestris. (TIF) [file pone.0154573.s002.tif]

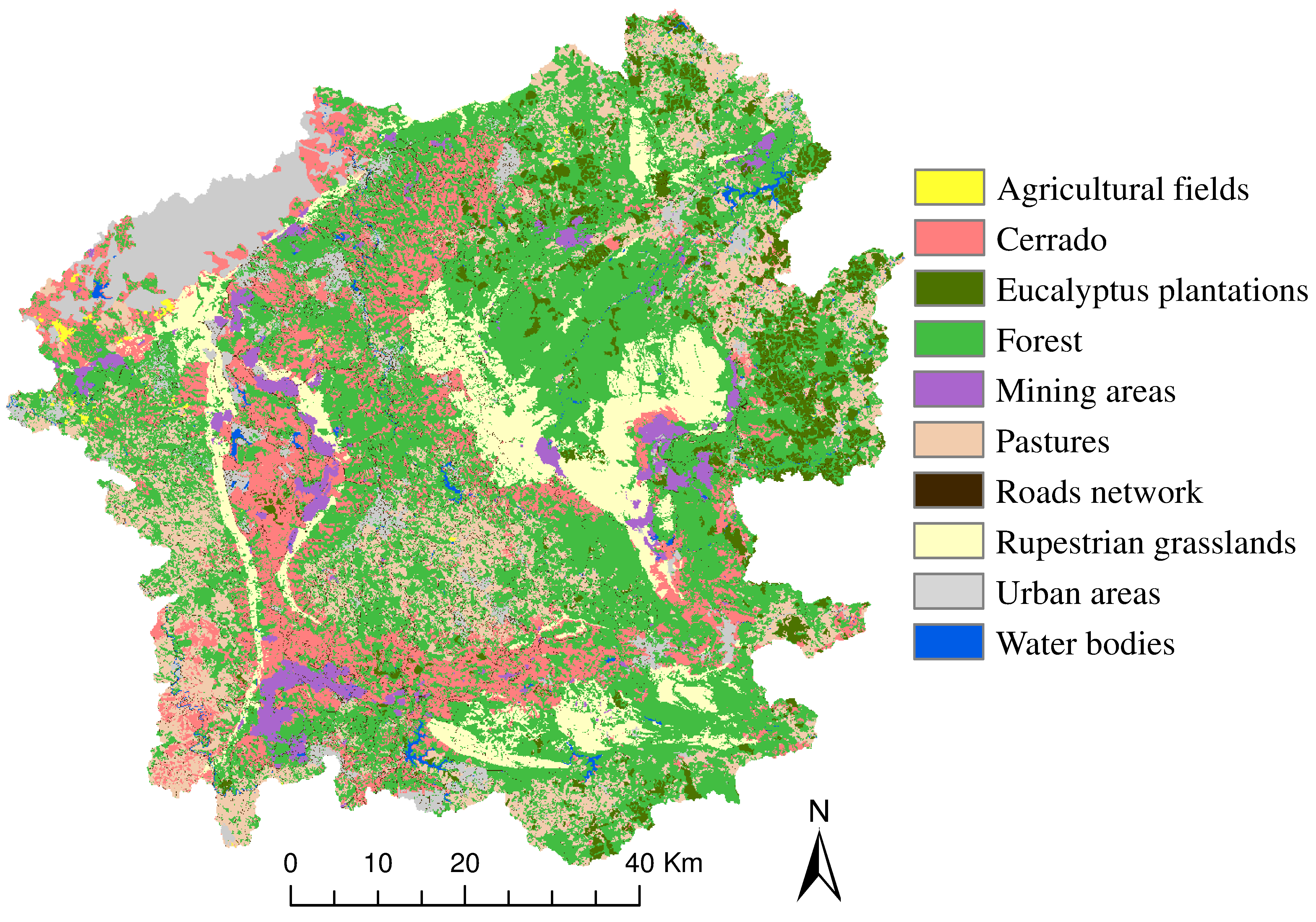

Supplement: S2 Fig — (TIF) [file pone.0154573.s003.tif]
